# Récidive d'un tératome immature de l'ovaire avec gliomatose péritonéale

**DOI:** 10.11604/pamj.2015.20.54.5056

**Published:** 2015-01-21

**Authors:** Houssine Boufettal, Naïma Samouh

**Affiliations:** 1 Service de Gynécologie-Obstétrique «C», Centra Hospitalier Universitaire Ibn Rochd, Faculté de Médecine et de Pharmacie, Université Hassan 2, Casablanca, Maroc

**Keywords:** Tératome immature, tératome mature, ovaire, gliomatose péritonéale, récidive, échographie, immature teratoma, mature teratoma, ovary, peritoneal gliomatosis, recurrence, ultrasound

## Abstract

Le tératome immature est une tumeur germinale non séminomateuse très rare, dont l’évolution est variable dans les différents cas publiés (récidive, maturation, dégénérescence). Nous rapportons un cas de récidive d'un tératome immature de l'ovaire sous forme d'un tératome mature, après traitement chirurgical conservateur et chimiothérapie. Il s'agit d'une patiente âgée de 25 ans, qui était opérée pour un tératome immature de l'ovaire avec gliomatose péritonéale par une annexectomie droite et des biopsies des granulations péritonéales ainsi qu'une chimiothérapie adjuvante, à base de BEP comprenant bléomycine, étoposide et un sel de platine (cisplatinum). Une rechute était notée 18 mois après le traitement sous forme d'un tératome mature de l'ovaire gauche. Avec un recule de 36 mois, aucune récidive n’était notée. La surveillance des tératomes ovariens associés à une gliomatose péritonéale doit être rapprochée et prolongée. En effet, les tératomes matures peuvent dégénérer ou récidiver. Les tératomes immatures peuvent récidiver au niveau de l'ovaire controlatéral sous forme mature ou immature. Les implants peuvent subir une régression fibreuse ou, exceptionnellement, dégénérer vers une glioblastomatose, de mauvais pronostic. L’échographie pelvienne couplée au doppler reste l'examen de choix pour les masses annexielles. Elle doit être répétée au moindre doute, et complétée par la tomodensitométrie chez ces patientes.

## Introduction

Le tératome immature est une tumeur germinale non séminomateuse très rare (moins de 100 cas décrits) [1]. L’évolution de ces tératomes reste variable dans les différents cas publiés (récidive, maturation, dégénérescence). Nous rapportons un cas de récidive d'un tératome immature de l'ovaire sous forme d'un tératome mature, après traitement chirurgical conservateur et chimiothérapie.

## Patient et observation

**Figure 1:**
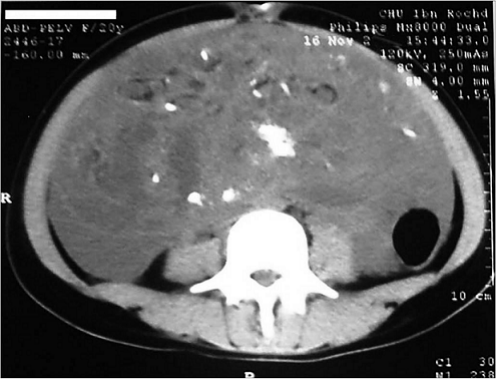
TDM abdominopelvienne montrant une volumineuse masse abdominopelvienne solidokystique

**Figure 2:**
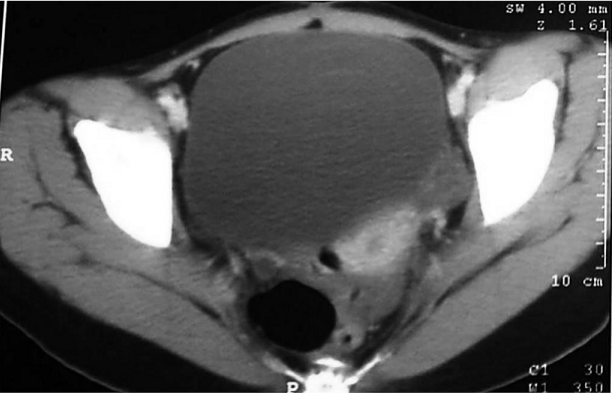
Tomodensitométrie abdominopelvienne de contrôle montrant réduction de cette masse à 14 mm

Une patiente âgée de 25 ans, était opérée il y a cinq ans pour un tératome immature de l'ovaire droit avec gliomatose péritonéale révélé par une importante masse abdomino-pelvienne augmentant rapidement de volume (Figure 1). Une annexectomie droite ainsi qu'une biopsie de granulations péritonéales étaient réalisées. L'examen anatomopathologique objectivait un tératome multitissulaire immature avec gliomatose péritonéale, de grade histo-pronostic 3 selon la classification de Norris et O'Connor et de stade 1a selon la FIGO associé à une gliomatose péritonéale au niveau des nodules péritonéaux. La patiente était mise sous six cycles de chimiothérapie adjuvante, instaurée deux mois après l'intervention, à base de BEP comprenant bléomycine, étoposide et un sel de platine (cisplatinum). Une surveillance mensuelle par l’échographie abdomino-pelvienne était réalisée ainsi qu'un dosage de l'AFP. Une échographie abdomino-pelvienne et une tomodensitométrie abdomino-pelvienne de contrôle, faite 12 mois après l'intervention, avaient montré une masse latéroutérine de 14 mm (Figure 2). Les marqueurs tumoraux (AFP et CA125) étaient négatifs. L'exploration chirurgicale montrait un nodule péritonéal. L'exérèse chirurgicale et l’étude anatomo-pathologique de ce nodule montraient qu'il s'agissait d'un tissu glial mature. Une échographie abdominopelvienne montrait, six mois après, une image une masse d’échostructure mixte solidokystique mesurant 52/49 mm. L'exploration chirurgicale montrait qu'il s'agissait d'une masse kystique au dépens de l'ovaire gauche, qui mesurait 55/50 mm. L'exploration de la cavité abdominopelvienne ne montrait pas, macroscopiquement, de nodules péritonéaux. Des biopsies péritonéales, pariétales et épiploïques étaient réalisées. L'examen anatomopathologique montrait qu'il s'agissait d'un tératome mature de l'ovaire gauche. Il n'y avait pas de lésions de malignité ou de gliomatose péritonéale sur les prélèvements péritonéaux réalisés. Avec un recule de 36 mois, aucune récidive n’était notée.

## Discussion

Le tératome immature de l'ovaire est une tumeur maligne composée de tissus dérivés des trois lignées cellulaires embryonnaires (le méso-, l'endo- et l'ectoderme) présents, à des stades de maturation différents, au sein de la tumeur. La quantité de tissu neural immature permet d’établir une classification en trois grades de malignité croissante. Les implants de tissu glial au niveau du péritoine réalisent la gliomatose péritonéale. Il touche fréquemment la femme jeune de la deuxième décade [1], comme c'est le cas de notre patiente. La tumeur est souvent unilatérale, de grande taille, arrondie, uni- ou polylobée et de composante mixte, liquide et solide [2]. Le pronostic initial est étroitement lié au grade histologique de la tumeur primitive et de ses implants. L'ovaire controlatéral présente un tératome mature dans 26% des cas et un autre tératome immature dans 10% des cas [1]. L'aspect à l'imagerie des tératomes immatures est peu spécifique, il est celui d'un tératome ovarien [3]. L’évolution rapide et la taille tumorale évoque plutôt le caractère immature. Les implants péritonéaux sont visibles macroscopiquement sous forme de petits nodules blanchâtres ou de découverte histologique sur des prélèvements péritonéaux. La gliomatose péritonéale accompagne le plus souvent des tératomes matures, plus rarement immatures [1–3]. Dans notre cas, le tératome était immature. Le traitement chirurgical constitue généralement le premier temps thérapeutique. Il consiste en une annexectomie initiale par laparotomie médiane permettant dans le même temps une exploration de la cavité abdominale avec prélèvement de liquide péritonéal pour étude cytologique et biopsies péritonéales multiples [1, 4].

La surveillance des tératomes ovariens associés à une gliomatose péritonéale doit être rapprochée et prolongée. En effet, les tératomes matures peuvent dégénérer ou récidiver dans 15 à 30% des cas. Les tératomes immatures peuvent récidiver au niveau de l'ovaire controlatéral sous forme mature ou immature [1, 5]. Dans notre observation, la récidive du tératome immature était sous forme mature, dix huit mois après l'annexectomie droite. La maturation des tératomes immatures semblerait être une propriété intrinsèque de ce type de tumeur. L'extension péritonéale se faisant par l'intermédiaire d'une brèche capsulaire avec maturation secondaire in situ [1, 3–5]. La possibilité d'une maturation, par l'intermédiaire des agents de chimiothérapie, a été évoquée. En effet, elle n'est généralement mise en évidence qu'après une chirurgie de second regard post chimiothérapie [1, 2, 6]. Plus inhabituel, il était rapporté un cas de tératome immature de l'ovaire, avec métastases hépatiques, devenu mature après chimiothérapie adjuvante évoluant vers un adénocarcinome 12 ans plus tard [2]. Aussi, les implants peuvent évoluer différemment. Ils peuvent rester asymptomatiques et stables, insensibles à la chimiothérapie ou subir une régression fibreuse, comme ils peuvent, exceptionnellement, dégénérer vers un glioblastome, réalisant une glioblastomatose. Cette dernière est associée à un mauvais pronostic [4, 7]. Les lésions secondaires des tératomes immatures, siégeant souvent, sur l'ensemble du péritoine abdominopelvien, parfois sur le grand épiploon ou d'autres organes comme le foie, sont généralement sous une forme immature, peuvent aussi subir une maturation de ces lésions secondaires (grade 0) dans 4 à 13% selon les auteurs [1, 8]. Le rôle de la chimiothérapie n'est pas clair dans cette transformation. L'alphafoetoprotéine est un marqueur sérique utilisé pour la surveillance des tumeurs à cellules germinales, mais sa sensibilité est médiocre et elle n'est pas spécifique des tératomes de grade élevé [1, 3, 9]. L’échographie pelvienne couplée au doppler reste l'examen de choix pour les masses annexielles. Ainsi, les échographies doivent être répétées au moindre doute, complétées par la tomodensitométrie chez les patientes porteuses d'une gliomatose disséminée.

## Conclusion

Le tératome immature est très rare. Son évolution après exérèse complète, est variable. Il peut se faire vers la récidive, la maturation ou la dégénérescence, justifiant une surveillance à long terme de ces cancers.
